# Carpal Tunnel Syndrome Attributed to Medication Use: A Pharmacovigilance Study

**DOI:** 10.7759/cureus.83972

**Published:** 2025-05-12

**Authors:** Andrew Mihalache, Emily Volfson, Ryan Huang, Kevin Zuo, Jonathan Persitz

**Affiliations:** 1 Medicine, Temerty Faculty of Medicine, University of Toronto, Toronto, CAN; 2 Hand Program, Toronto Western Hospital, University Health Network, Toronto, CAN

**Keywords:** carpal tunnel syndome, drugs, faers, neuropathy, pharmacovigilance

## Abstract

Objective: Carpal tunnel syndrome (CTS) is a prevalent compression neuropathy with multiple well-documented mechanical and systemic risk factors. However, the role of pharmacological agents in the development of CTS remains underexplored. This study aims to identify drugs disproportionately associated with CTS reports using data from the Food and Drug Administration Adverse Event Reporting System (FAERS).

Methods: A retrospective pharmacovigilance analysis was conducted using OpenVigil 2.1 to evaluate adverse event (AE) reports of CTS from the FAERS database from October 2003 to September 2024. Only drugs identified as the primary suspect in at least 10 AE reports were included. Disproportionality analysis, including reporting odds ratios (RORs), was used to assess associations between CTS and specific drugs. Positive signals were validated using Bayesian confidence propagation neural network algorithms, with drugs having ROR ≥10 and significant Bayesian confidence intervals (IC025 > 0) considered strongly associated with CTS.

Results: Of 12,929,504 AEs reported during the study period, 6,837 (0.05%) involved CTS. Female patients comprised 69.5% of CTS cases, with a mean age of 57.0±14.9 years. Ten drugs were found to have significant overreporting of CTS, including idursulfase (ROR=51.2, 95% CI=39.0-67.2), galsulfase (ROR=26.8, 95% CI=17.2-41.7), laronidase (ROR=20.9, 95% CI=14.4-30.3), tesamorelin (ROR=20.7, 95% CI=13.7-31.3), anastrozole (ROR=20.6, 95% CI=17.0-24.9), alendronic acid (ROR=17.1, 95% CI=14.5-20.1), gamma-hydroxybutyric acid (GHB) (ROR=16.3, 95% CI=9.6-27.6), rofecoxib (ROR=16.1, 95% CI=14.3-18.2), alendronate (ROR=12.9, 95% CI=11.0-15.2), and tafamidis (ROR=12.0, 95% CI=9.2-15.7).

Conclusions: Several drugs were disproportionately associated with CTS in the FAERS database, including enzyme replacement therapies (ERTs), aromatase inhibitors, bisphosphonates, growth hormone (GH)-releasing factor analogs, GHB, rofecoxib, and tafamidis. These findings highlight the critical need for increased vigilance and monitoring of new-onset or worsening CTS in high-risk populations prescribed the aforementioned medications. Clinicians should carefully scrutinize pharmacological history when evaluating patients in this context.

## Introduction

Carpal tunnel syndrome (CTS), the most common compression neuropathy of the upper extremity, arises from the compression of the median nerve as it traverses the carpal tunnel in the wrist [1]. In the United States, CTS is reported to have an incidence of one to three cases per 1,000 individuals and a prevalence of about 50 cases per 1,000 people [1]. The pathophysiology involves increased carpal tunnel pressure, caused either by surrounding structures or by edema of the median nerve, leading to ischemic injury and impaired signal conduction [2]. Classic symptoms of CTS include pain, paresthesia, loss of dexterity, and eventually, hand weakness [1]. Depending on its severity, CTS may significantly compromise hand functionality and impact the quality of life [1]. Women and older adults are disproportionately affected, as hormonal changes and age-related factors are key contributors to its pathophysiology [3]. Various etiological factors, including repetitive extreme wrist movements, anatomical anomalies, systemic inflammatory conditions, and metabolic disorders such as diabetes mellitus and hypothyroidism, are recognized risk factors for CTS [1,4].

Despite extensive documentation of mechanical and systemic risk factors for CTS [5,6], there is a paucity of research exploring the role of pharmacological agents in this context [7,8]. A more nuanced understanding of drug-related risks is critical, as such insights may enable more tailored, patient-centered therapies. Importantly, the discontinuation or substitution of implicated medications has the potential to alleviate CTS symptoms and mitigate the necessity for surgical intervention. To address this gap, this study represents the first comprehensive pharmacovigilance analysis, to the best of our knowledge, to identify associations between specific drugs and CTS. By analyzing data from the Food and Drug Administration Adverse Event Reporting System (FAERS), we aim to uncover drugs disproportionately linked to reported cases of CTS, providing critical insights for preventive strategies and optimizing clinical decision-making [9].

## Materials and methods

Our retrospective pharmacovigilance analysis utilized OpenVigil 2.1 (Kiel, Germany) data-mining software to evaluate adverse event (AE) reports of CTS documented in the FAERS [9] from October 2003 to September 2024. The Medical Dictionary for Regulatory Activities (MedDRA) Preferred Term (PT) used to query OpenVigil 2.1 was "carpal tunnel syndrome.”

This study did not require institutional review board approval as it used only publicly available, de-identified data. Moreover, this study was conducted in accordance with the tenets of the Declaration of Helsinki.

Descriptive statistics were employed to summarize patient characteristics, including age, gender, continent, and reporting year. Disproportionality analyses involving reporting odds ratios (RORs) were conducted to investigate associations between CTS reports and specific pharmacological agents. RORs were calculated using the formula

$\text{ROR} = \frac{a/c}{b/d}$

where a represents the number of reports pertaining to both the drug of interest and CTS, b represents the number of reports pertaining to the drug of interest and all other AEs (excluding CTS), c represents the number of reports pertaining to all other drugs (excluding the drug of interest) and CTS, and d represents the number of reports pertaining to all other drugs (excluding the drug of interest) and all other AEs (excluding CTS) [10]. As such, this approach compares the odds of CTS cases attributed to each drug against all other drugs in the FAERS.

Positive signals for adverse drug reactions were identified using the methodology outlined by Evans et al. (2001), which requires significant drug-reaction pairs to meet the following criteria: (i) at least three reported cases; (ii) a proportional reporting ratio (PRR) of at least two, where $\text{PRR} = \frac{a/(a + b)}{c/(c + d)}$

and (iii) a chi-squared value of at least four [11]. These signals were validated using Bayesian confidence propagation neural network algorithms [12], where a drug was deemed to have an overreported association with CTS if the lower bound of the 95% confidence interval (CI) for the information component (IC) exceeded zero (IC025 > 0) [12]. To further mitigate the risk of false positive associations, only drugs identified as the primary suspect in ≥10 CTS reports and those with an ROR of ≥10 were considered. These custom thresholds were applied to effectively filter out pharmacological agents in the FAERS that may have exhibited a statistically significant yet potentially spurious safety signal, considering their relatively low absolute number of cases attributed to CTS.

## Results

**Figure 1:**
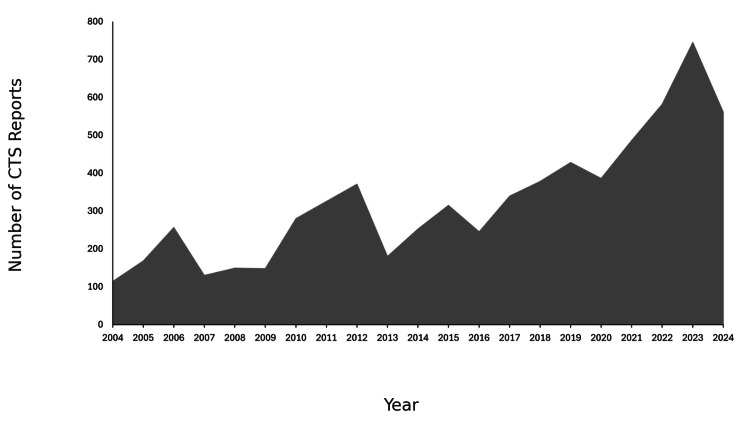
Number of CTS reports over the study period CTS: carpal tunnel syndrome

During the study period, a total of 12,929,504 AEs were reported to the FAERS, of which 6,837 (0.05%) involved CTS. Among these, 4,753 reports (69.5%) were from females, 1,717 (25.1%) were from males, and 367 (5.4%) did not specify gender. The mean age of the reported cases was 57.0±14.9 years, with the majority originating from the United States (n=4,699, 68.7%). A summary of the annual number of CTS cases reported between 2004 and 2024 is provided in Figure 1.

Several drugs were found to be significantly overreported in association with CTS, including idursulfase (n=54 of 2,064, 2.6%; ROR=51.2, 95% CI=39.0-67.2), galsulfase (n=20 of 1,435, 1.4%; ROR=26.8, 95% CI=17.2-41.7), laronidase (n=28 of 2,577, 1.1%; ROR=20.9, 95% CI=14.4-30.3), tesamorelin (n=23 of 2,130, 1.1%; ROR=20.7, 95% CI=13.7-31.3), anastrozole (n=109 of 10,273, 1.1%; ROR=20.6, 95% CI=17.0-24.9), alendronic acid (n=151 of 17,251, 0.9%; ROR=17.1, 95% CI=14.5-20.1), gamma-hydroxybutyric (GHB) acid (n=14 of 1,640, 0.9%; ROR=16.3, 95% CI=9.6-27.6), rofecoxib (n=268 of 32,890, 0.8%; ROR=16.1, 95% CI=14.3-18.2), alendronate (n=157 of 23,592, 0.7%; ROR=12.9, 95% CI=11.0-15.2), and tafamidis (n=55 of 8,770, 0.6%; ROR=12.0, 95% CI=9.2-15.7).

Table 1 summarizes the drugs most disproportionately associated with CTS reports in the FAERS database, including the number and percentage of CTS cases, RORs, and 95% CIs.

**Table 1:** Ten drugs most strongly associated with CTS in FAERS, with case counts, RORs, and 95% CIs CTS: carpal tunnel syndrome; FAERS: Food and Drug Administration Adverse Event Reporting System; ROR: reporting odds ratio; CI: confidence interval

Drug	CTS reports (n of N, %)	Reporting odds ratio (ROR)	95% confidence interval (CI)
Idursulfase	54 of 2,064 (2.6%)	51.2	39.0-67.2
Galsulfase	20 of 1,435 (1.4%)	26.8	17.2-41.7
Laronidase	28 of 2,577 (1.1%)	20.9	14.4-30.3
Tesamorelin	23 of 2,130 (1.1%)	20.7	13.7-31.3
Anastrozole	109 of 10,273 (1.1%)	20.6	17.0-24.9
Alendronic acid	151 of 17,251 (0.9%)	17.1	14.5-20.1
Gamma-hydroxybutyric acid	14 of 1,640 (0.9%)	16.3	9.6-27.6
Rofecoxib	268 of 32,890 (0.8%)	16.1	14.3-18.2
Alendronate	157 of 23,592 (0.7%)	12.9	11.0-15.2
Tafamidis	55 of 8,770 (0.6%)	12.0	9.2-15.7

**Figure 2:**
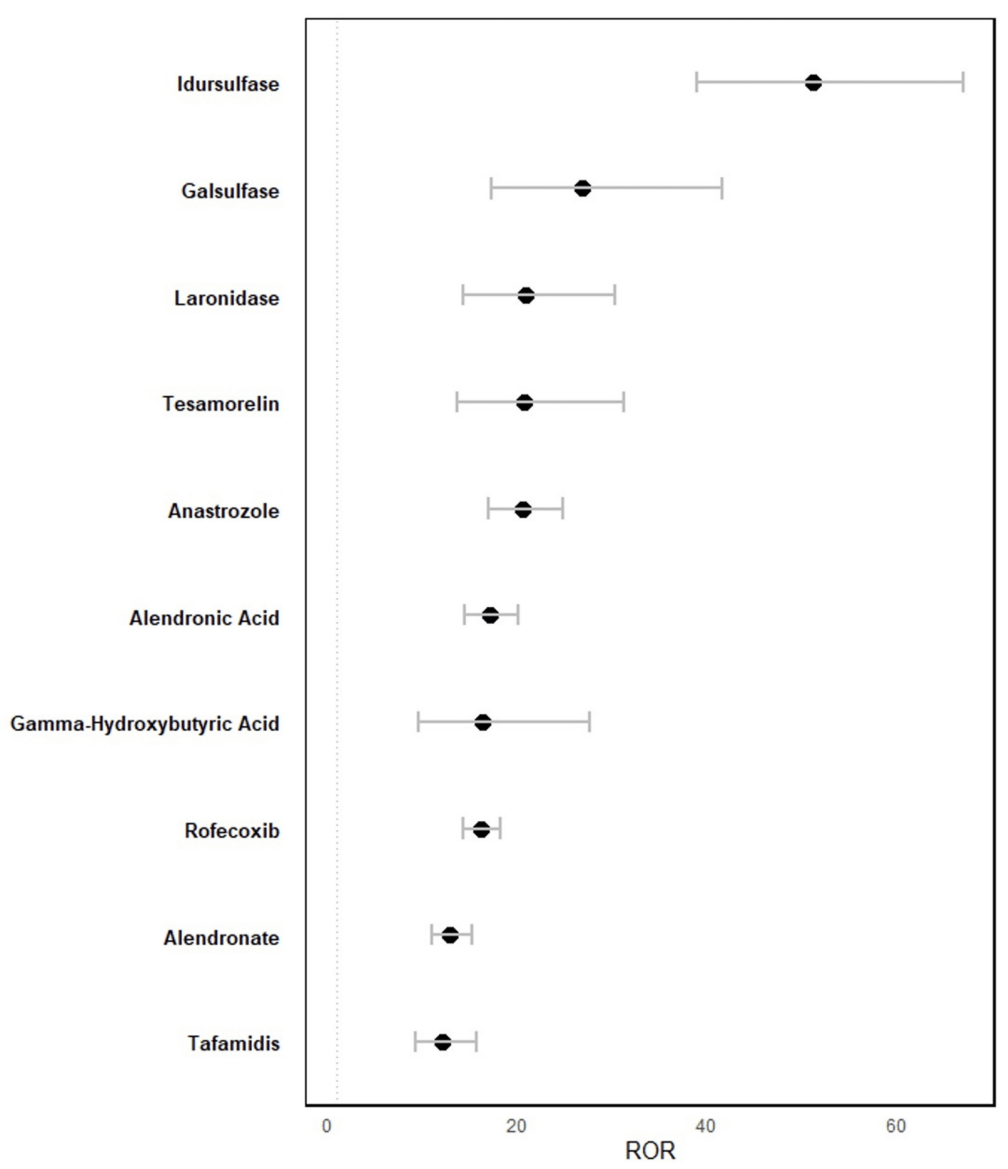
RORs of primary suspect drugs attributed to reports of carpal tunnel syndrome ROR: reporting odds ratio

A summary of the overreported drugs is depicted in Figure 2. Additionally, Table 2 presents a summary of their indications, routes of administration, and mechanisms of action.

**Table 2:** Summary of the most overreported drugs in association with carpal tunnel syndrome GABA: gamma-aminobutyric acid

Drug	Primary indication	Route of administration	Mechanism
Idursulfase	Mucopolysaccharidosis II	Intravenous	Replaces iduronate-2-sulfatase enzyme
Galsulfase	Mucopolysaccharidosis I	Intravenous	Replaces N-acetylgalactosamine-4-sulfatase enzyme
Laronidase	Mucopolysaccharidosis VI	Intravenous	Replaces α-L-iduronidase enzyme
Tesamorelin	Acquired lipodystrophy	Subcutaneous	Stimulates growth hormone release
Gamma-hydroxybutyric acid	Narcolepsy	Oral	Modulates GABA receptors and reduces cataplexy
Anastrozole	Breast cancer	Oral	Inhibits aromatase to reduce estrogen production
Tafamidis	Cardiac amyloidosis	Oral	Stabilizes transthyretin to prevent misfolding
Alendronic acid	Osteoporosis	Oral	Inhibits osteoclast-mediated bone resorption
Alendronate	Osteoporosis	Oral	Inhibits osteoclast-mediated bone resorption

## Discussion

To the best of our knowledge, this study represents the first comprehensive pharmacovigilance analysis to identify associations between specific drugs and CTS. By leveraging an extensive pharmacovigilance database, our findings offer novel insights into potential drug-induced mechanisms underlying CTS and emphasize the importance of monitoring for this condition in certain patient populations. Notably, medications such as idursulfase, galsulfase, laronidase, tesamorelin, anastrozole, alendronic acid, GHB, rofecoxib, alendronate, and tafamidis exhibited a disproportionately high reporting of CTS. While these findings merit further validation through large-scale prospective studies, they underscore potential iatrogenic factors that clinicians must consider when prescribing these medications. In rare instances, inflammatory complications such as necrotizing granulomatous inflammation following carpal tunnel surgery have also been described, underscoring the importance of considering atypical postoperative responses in the broader pharmacovigilance landscape [13].

Idursulfase emerged as the most disproportionately overreported drug for CTS in our pharmacovigilance analysis. However, this association was likely driven by the underlying disease pathology rather than the treatment itself. As an enzyme replacement therapy (ERT), idursulfase is often prescribed for mucopolysaccharidosis (MPS) II (Hunter syndrome), facilitating the degradation of glycosaminoglycans (GAGs) by replacing the deficient enzyme iduronate-2-sulfatase [14]. While idursulfase is designed to reduce systemic GAG accumulation, localized deposits of GAGs in connective tissues, including the carpal tunnel, often persist or progress despite therapy. For instance, CTS is notably prevalent in patients with MPS I and II, with studies reporting rates as high as 90%, whereas it is rarely observed in MPS III [15]. The strong safety signal observed in our study suggests the possibility of CTS exacerbations in patients receiving idursulfase. Given the disproportionately high number of primary suspect reports submitted to the FAERS implicating idursulfase in cases of CTS, future research is warranted to determine whether idursulfase might paradoxically contribute to CTS through mechanisms that have not yet been elucidated.

Similar to idursulfase, a disproportionately high number of cases of CTS have been reported wherein galsulfase or laronidase were identified as primary suspect drugs. Galsulfase and laronidase are other ERTs used to manage MPS [16]. Galsulfase, indicated for Maroteaux-Lamy syndrome (MPS VI), addresses the deficiency of arylsulfatase B; laronidase, used for Hurler, Hurler-Scheie, and Scheie syndromes (MPS I), replaces the enzyme alpha-L-iduronidase [16].

In both MPS I and VI, GAG deposition in the subsynovial connective tissues of the carpal tunnel can increase intracanal pressure, leading to median nerve compression and the development of CTS [15,17]. Nonetheless, it is plausible that in patients who do not exhibit CTS prior to initiating ERT, disease progression, rather than the treatment itself, may be the primary driver of CTS.

Tesamorelin, a growth hormone (GH)-releasing factor analog, is primarily used to reduce visceral adiposity in patients with HIV-associated lipodystrophy [18]. Tesamorelin stimulates the pituitary gland, thereby increasing the secretion of endogenous GH, which subsequently elevates insulin-like growth factor 1 (IGF-1) levels [19]. Elevated levels of GH and IGF-1 may lead to soft tissue swelling, increased extracellular matrix production, and sodium and water retention, all of which can potentially narrow the carpal tunnel space and compress the median nerve [20]. Despite there being a paucity of tesamorelin-induced CTS reports in the literature, GH therapy has been associated with CTS in other contexts [21]. For instance, a case report documented bilateral median neuropathy in a patient undergoing GH treatment, with symptoms resolving upon discontinuation of therapy, highlighting a direct link between elevated GH levels and CTS development [21].

Anastrozole, a third-generation aromatase inhibitor, is widely used in the adjuvant treatment of hormone receptor-positive breast cancer [22]. Aromatase inhibitors have been reported to increase the risk of CTS by inducing inflammation and edema in the flexor compartment of the wrist [23]. In a double-blind randomized controlled trial, Spagnolo et al. (2016) explored the association between anastrozole and CTS, demonstrating that 3.4% of women in the anastrozole group developed CTS, compared to 1.6% in the placebo group (p<0.001) [24]. Severe CTS requiring surgical intervention was also more common with anastrozole, occurring in 0.9% of cases versus 0.3% in the placebo group (p=0.018) [24]. More recently, Chien et al. (2020) found a higher one-year cumulative incidence of CTS in women with breast cancer using aromatase inhibitors (1.4%) versus tamoxifen (0.8%; p=0.008), with a 68% increased CTS risk for aromatase inhibitor users in multivariable analysis [25].

Alendronic acid is a bisphosphonate commonly prescribed for osteoporosis and other metabolic bone disorders [26]. Carvajal et al. (2016) reported a higher CTS incidence in postmenopausal women using bisphosphonates (1.2%) versus non-users (0.8%), with no significant risk differences across exposure levels, indicating even minimal exposure may increase risk [27]. These findings underscore the importance of monitoring for CTS symptoms in patients receiving bisphosphonate therapy, particularly postmenopausal women, who may already be at higher baseline risk.

GHB is a therapeutic agent used in the treatment of narcolepsy, effectively reducing cataplexy episodes and excessive daytime sleepiness [28]. While the association between GHB and CTS remains poorly documented, indirect evidence supports a potential link. GHB-induced sedation may lead to prolonged immobility, with the wrist positioned in hyperflexion or hyperextension, increasing carpal tunnel pressure and compressing the median nerve, potentially linking GHB to CTS [29]. Additionally, GHB has been shown to affect muscle tone and nerve conductivity, potentially contributing to peripheral neuropathy [30]. Further research is necessary to elucidate the mechanisms by which GHB might contribute to CTS development, as current evidence remains limited and indirect.

Tafamidis is a transthyretin stabilizer indicated for the treatment of transthyretin amyloid cardiomyopathy [31]. Transthyretin amyloidosis is characterized by amyloid deposition in various tissues, including peripheral nerves [32]. As such, CTS is a common manifestation in patients with amyloid neuropathy [33]. The occurrence of CTS in patients receiving tafamidis likely reflects the underlying disease pathology rather than a direct effect of the drug, albeit future research is warranted to clarify this relationship.

Our study had several limitations inherent to the use of data from the FAERS. As the FAERS relies on voluntary reporting of AEs, underreporting or selective reporting of certain AEs may have introduced biases that affected the robustness of our findings. Additionally, the observational nature of this pharmacovigilance analysis limits our ability to establish causal relationships between specific drugs and CTS. Patient-specific factors, including comorbidities, occupational risks, concomitant medication use, and drug dosing, could not be accounted for due to the lack of detailed clinical data in AE reports. Confounding by indication is another potential limitation, as certain drugs identified as primary suspects for CTS (i.e., idursulfase, galsulfase, and laronidase) are often prescribed to populations already at an elevated risk of developing CTS due to underlying conditions. This confounding factor makes it challenging to determine whether the associations are attributable to the drugs themselves or to the diseases they are used to treat.

## Conclusions

This pharmacovigilance analysis identified several drugs disproportionately associated with CTS in the FAERS database, including ERTs, aromatase inhibitors, bisphosphonates, GH-releasing factor analogs, GHB, rofecoxib, and tafamidis. It remains unclear whether some of the aforementioned drugs may have contributed to CTS via mechanisms related to their pharmacological effects or the underlying conditions for which they were prescribed. Future prospective studies are needed to validate these associations and develop strategies to mitigate CTS risk.
